# Stereotactic radioablation for the treatment of ventricular tachycardia: preliminary data and insights from the STRA-MI-VT phase Ib/II study

**DOI:** 10.1007/s10840-021-01060-5

**Published:** 2021-10-05

**Authors:** Corrado Carbucicchio, Daniele Andreini, Gaia Piperno, Valentina Catto, Edoardo Conte, Federica Cattani, Alice Bonomi, Elena Rondi, Consiglia Piccolo, Sabrina Vigorito, Annamaria Ferrari, Matteo Pepa, Mattia Giuliani, Saima Mushtaq, Antonio Scarà, Leonardo Calò, Alessandra Gorini, Fabrizio Veglia, Gianluca Pontone, Mauro Pepi, Elena Tremoli, Roberto Orecchia, Giulio Pompilio, Claudio Tondo, Barbara Alicja Jereczek-Fossa

**Affiliations:** 1 Department of Clinical Electrophysiology and Cardiac Pacing, Centro Cardiologico Monzino IRCCS, Via Carlo Parea 4, 20138 Milan, Italy; 2 Department of Cardiovascular Imaging, Centro Cardiologico Monzino IRCCS, Milan, Italy; 3 Department of Clinical Sciences and Community Health, University of Milan, Milan, Italy; 4 Division of Radiotherapy, IEO European Institute of Oncology IRCCS, Milan, Italy; 5 Unit of Medical Physics, IEO European Institute of Oncology IRCCS, Milan, Italy; 6 Biostatistics Unit, Centro Cardiologico Monzino IRCCS, Milan, Italy; 7 Psycho-Cardiology Service, Centro Cardiologico Monzino IRCCS, Milan, Italy; 8 Unit of Cardiology, Policlinico Casilino, Rome, Italy; 9 Department of Oncology and Hemato-Oncology, University of Milan, Milan, Italy; 10 Clinical Area Directorate, Centro Cardiologico Monzino IRCCS, Milan, Italy; 11 Prevention Program Directorate, Centro Cardiologico Monzino IRCCS, Milan, Italy; 12 Scientific Directorate, IEO European Institute of Oncology IRCCS, Milan, Italy; 13 Scientific Directorate, Centro Cardiologico Monzino IRCCS, Milan, Italy; 14 Department of Biomedical, Surgical and Dental Sciences, University of Milan, Milano, Italy

**Keywords:** Ventricular tachycardia/ventricular arrhythmias, Stereotactic body radiotherapy/radioablation, Catheter ablation, Structural heart disease/dilated cardiomyopathy, Multimodal imaging

## Abstract

**Purpose:**

We present the preliminary results of the STRA-MI-VT Study (NCT04066517), a spontaneous, phase Ib/II study, designed to prospectively test the safety and efficacy of stereotactic body radiotherapy (SBRT) in patientswith advanced cardiac disease and intractable ventricular tachycardia (VT).

**Methods:**

Cardiac computed tomography (CT) integrated by electroanatomical mapping was used for substrate identification and merged with dedicated CT scans for treatment plan preparation. A single 25-Gy radioablation dose was delivered by a LINAC-based volumetric modulated arc therapy technique in a non-invasive matter. The primary safety endpoint was treatment-related adverse effects during acute and long-term follow-up (FU), obtained by regular in-hospital controls and implantable cardioverter defibrillator (ICD) remote monitoring. The primary efficacy endpoint was the reduction at 3 and 6 months of VT episodes and ICD shocks.

**Results:**

Seven out of eight patients (men; age, 70 ± 7 years; ejection fraction, 27 ± 11%; 3 ischemic, 4 non-ischemic cardiomyopathies) underwent SBRT. At a median 8-month FU, no treatment-related serious adverse event occurred. Three patients died from non-SBRT-related causes. Four patients completed the 6-month FU: the number of VT decreased from 29 ± 33 to 11 ± 9 (*p* = .05) and 2 ± 2 (*p* = .08), at 3 and 6 months, respectively; shocks decreased from 11 to 0 and 2, respectively. At 6 months, all patients. showed a significant reduction of VT episodes and no electrical storm recurrence, with the complete regression of iterative VTs in 2/2 patients.

**Conclusion:**

The STRA-MI-VT Study suggests that SBRT can be considered an alternative option for the treatment of VT in patients with structural heart disease and highlights the need for further clinical investigation addressing safety and efficacy.

**Supplementary Information:**

The online version contains supplementary material available at 10.1007/s10840-021-01060-5.

## Introduction

Radiofrequency catheter ablation (RFCA) has evolved into a first-line modality of therapy for patients with scar-related ventricular arrhythmias (VAs) [1–3], being effective in the prevention of ventricular tachycardia (VT) recurrences and in the reduction of implantable cardioverter defibrillator (ICD) interventions [4, 5]; furthermore, RFCA has been recognized as the treatment of choice in the management of incessant VT and electrical storm (ES), where the patient is exposed to a high risk of cardiac death [6].

The conventional catheter-based approach is, however, limited mainly in patients with non-ischemic cardiomyopathies, in whom the complexity and location of the arrhythmogenic scar [7, 8] critically favor the persistence of the arrhythmic condition. Moreover, the possibility of treating the epicardial aspect of the heart is frustrated in the case of prior cardiac surgery, and even the endocardial access to the ventricles may be limited by the presence of a mechanical prosthesis. Of note, VT RFCA is a complex procedure and exposes the patient with advanced cardiomyopathy to several potential complications, whereas in case of significant comorbidities, the high procedural risk may even constitute a contraindication for any interventional approach, with a negative prognostic impact.

Single-session high-dose stereotactic body radiotherapy (SBRT) has emerged as an alternative option for the treatment of refractory VT, with the potential to overcome limitations of conventional RFCA [9–15]. This non-invasive approach eliminates the procedural risk during both mapping and ablation phases and allows the delivery of energy to any theoretical target without the need for anatomical contact. We present the preliminary safety and efficacy results of a phase Ib/II study where SBRT was applied in a cohort of patients with advanced cardiomyopathy as bailout treatment for refractory VTs.

## Methods

STRA-MI-VT is a spontaneous, prospective, single-arm, phase Ib/II single-center study. The design of the study has been detailed elsewhere [16]*.* Fifteen patients are expected to be enrolled and final results will be available after September 2022.

### Patient population

Patients were selected from the population admitted at the Centro Cardiologico Monzino IRCCS, Milan, Italy, as the nationwide referral center for VT ablation; SBRT was carried out at the Istituto Europeo di Oncologia IRCCS, Milan, Italy. Each patient case was discussed by a multidisciplinary institutional expert panel to exclude other treatment options and confirm eligibility.

#### Enrollment criteria

Patients were included if they had a structural heart disease and relapsing VT (≥ 3 VT episodes conditioning ICD intervention) refractory to any form of pharmacological and non-pharmacological therapy, and *either* showed a contraindication to conventional RFCA, in relation to the high risk associated with the procedure, *or*, *alternatively*, were not suitable for any interventional or surgical approach.

Additional inclusion criteria were the following: left ventricle (LV) ejection fraction (EF) ≥ 20%; age ≥ 50 years; the presence of an ICD or subcutaneous ICD; and informed consent signed.

Exclusion criteria were the following: previous thoracic radiotherapy; active myocardial ischemia; cardiac revascularization < 120 days; NYHA class IV; life expectancy < 1 year, or other major contraindication.

### Safety and efficacy endpoints

#### The primary safety endpoint

*The primary safety endpoint* was represented by safety of SBRT during the early first month phase after SBRT and at the 3-, 6-, and 12-month follow-up (FU). Adverse effects were classified according to the International Common Terminology Criteria for Adverse Events v 5.0 / CTCAE document.

The following were considered serious adverse events:

*Death; cardio-circulatory arrest; acute myocardial infarction; cardiogenic shock; pericarditis, myocarditis, and/or endocarditis; acute heart failure; gastro-esophageal lesion; bronchopulmonary infection; injury to the respiratory system; and radio-induced oncogenesis.*

#### Primary efficacy endpoint

*The primary efficacy endpoint* was represented by the total number of VT/ventricular fibrillation (VF) episodes detected by the ICD at 3, 6, and 12 months, compared to the 3-month period preceding SBRT.

We identified the following:

*Number of VT/VF episodes causing antitachycardia pacing (ATP); number of VT/VF episodes causing ICD shock; total number of ICD shocks; and number of VT episodes below the tachycardia detection interval (TDI)*. According to literature [11, 12, 15], a 6-week “blanking period” after SBRT was taken into account in the analysis of VT recurrences. As a rule, pharmacological therapy, including antiarrhythmic drugs, was maintained the same as before SBRT and changes were determined only by specific clinical reasons.

#### Secondary endpoints

Secondary endpoints, evaluated at 3, 6, and 12 months, were as follows:

*Global mortality (at 12 months); variations in quality of life (QoL), using the SF-36 Health Questionnaire *[17]*; and changes in cardiac function assessed by LV EF on echocardiographic examination.*

### Interventional workflow

**Fig. 1:**
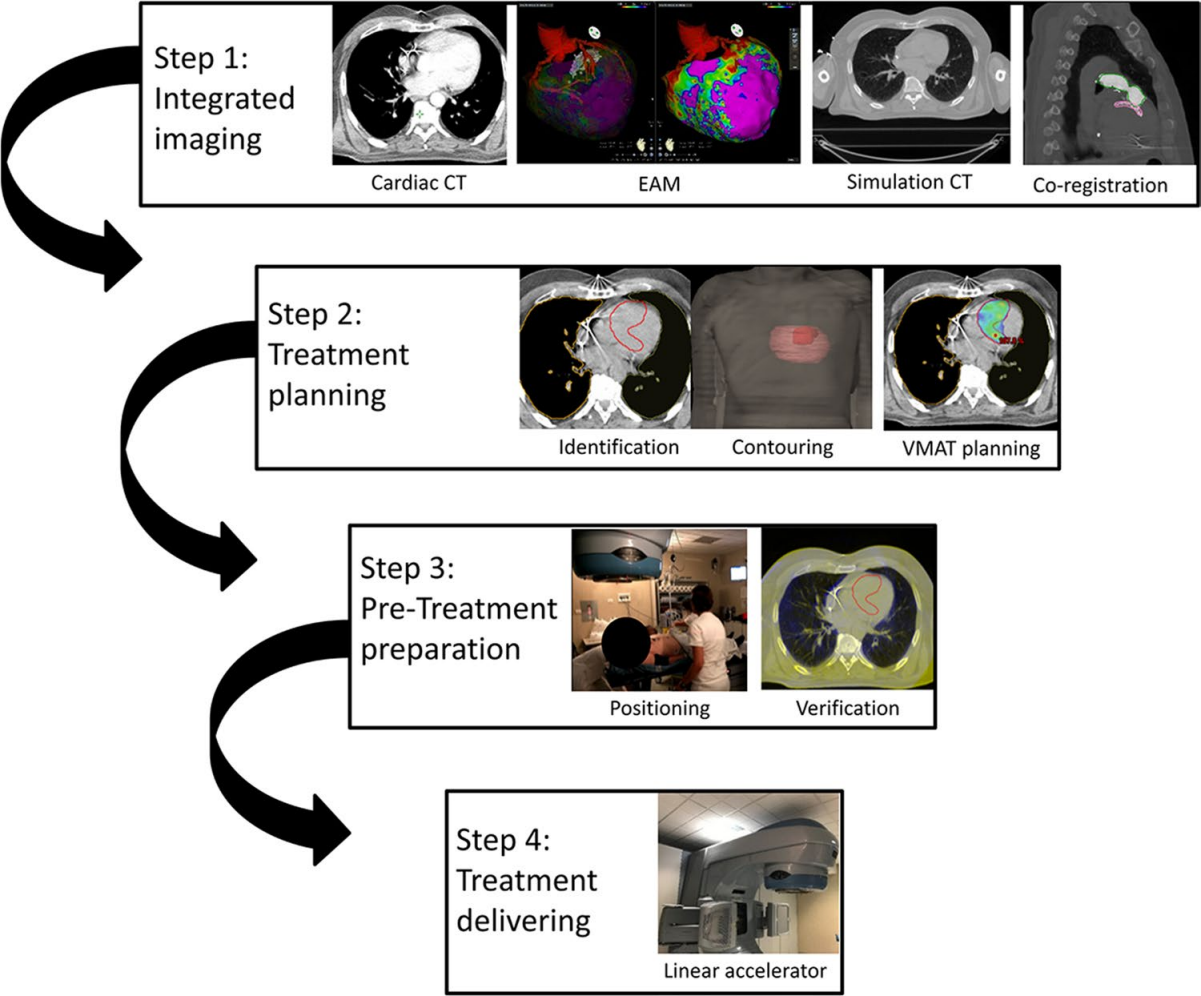
Interventional workflow. Step 1—cardiac CT data, integrated with endo-epicardial electroanatomical mapping, are merged with simulation “free-breathing” CT, acquired together with a “breathing-triggered” 4D-CT. Step 2—integrated “free-breathing” simulation-CT imaging is used as platform for the identification and contouring of the clinical target volume and of the organs at risk. The volumetric modulated arc therapy treatment plan is processed by the Eclipse RapidArc Planning System to deliver a single-fraction total dose of 25 Gy. Step 3—patient’s positioning setup is ensured by means of a vacuum immobilization cast on the treatment couch and is verified during the whole treatment process; two to three cone-beam CT scans are performed (image-guided radiotherapy), if necessary, for setup optimization. Step 4—the volumetric modulated arc therapy treatment is eventually delivered using the Varian Trilogy linear accelerator with the patient in the conscious state, lying down in a comfortable position in his/her immobilization cast. *Abbreviation:* CT, computed tomography

Electroanatomical mapping (EAM) in combination with delayed enhancement (DE) cardiac computed tomography (CT) was employed in as many patients as possible to characterize the arrhythmia substrate and to define the target area (Fig. 1). A 12-lead ECG analysis of all documented VTs was implemented to identify the VT breakthrough based on the QRS pattern. Non-invasive electrocardiographic imaging [18] (ECGI, EP Solutions SA) was achieved in selected cases. Detailed methods are reported in the Supplementary data.

#### Cardiac computed tomography

**Fig. 2:**
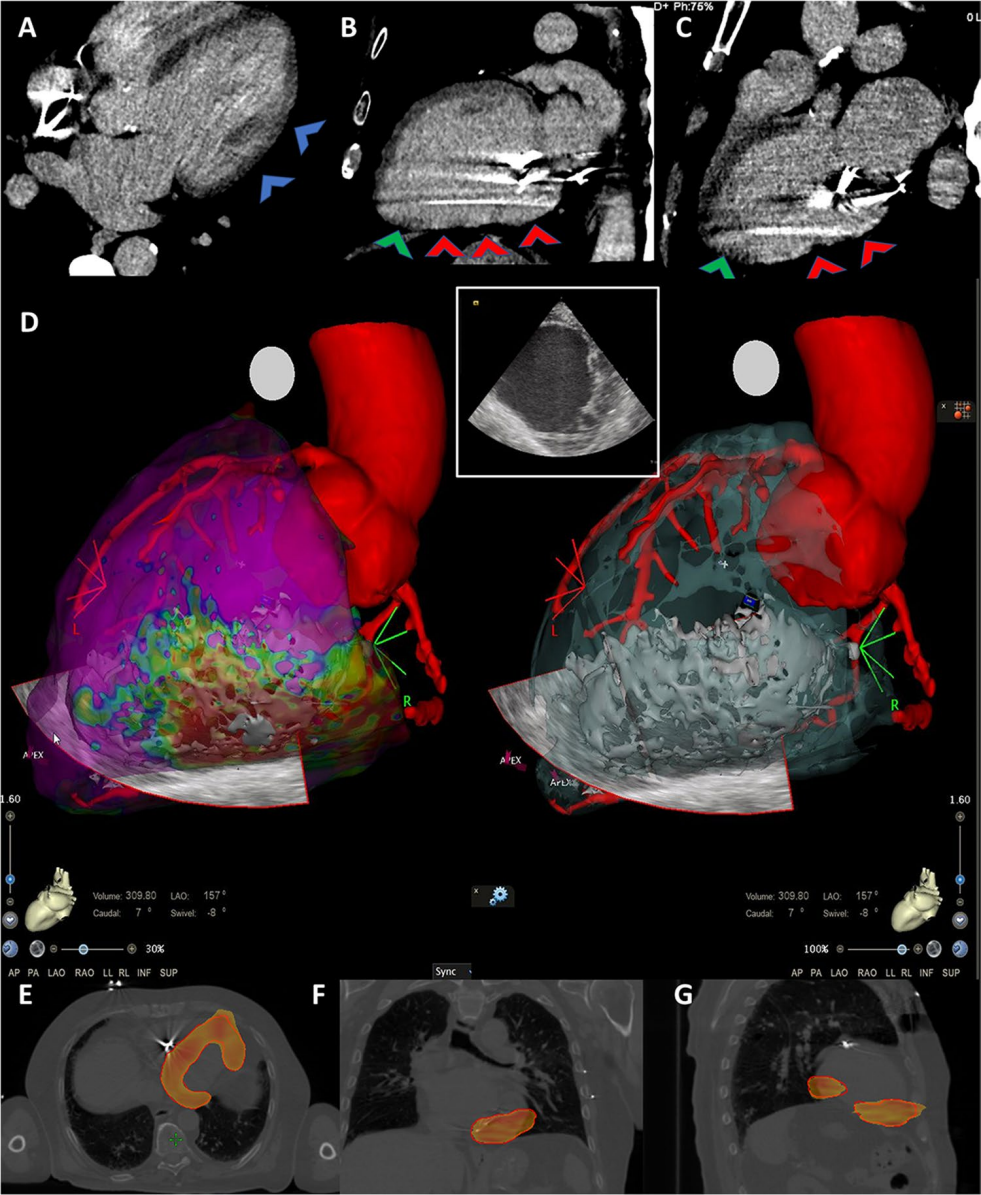
CT imaging with myocardial fibrosis evaluation, integrated electroanatomical mapping, and SBRT-treatment plan in one patient undergoing SBRT are represented. *Upper panels.* CT myocardial fibrosis evaluation in the long-axis view is represented. CT scan was acquired 8 min after the injection of iodinated contrast medium, as per protocol indication. No hyperdense myocardial areas are evident with regard to the anterolateral wall (**A**, blue arrowheads). Areas of hyperdense myocardium are shown (**B**, **C**): red arrowheads point at a transmural (ischemic pattern) myocardial fibrosis in the inferior and posterolateral wall of the left ventricle. The left ventricular apex appears free from lesions (green arrowheads). *Middle panel.* High-density epicardial electroanatomical mapping combined with CT imaging (**D**). Mid- and basal segments of the inferior and posterior wall are covered by diseased electrograms, represented in red and yellow by the “color-coded” map, as expression of the underlying electrical scar (left). In the same location, CT shows a pattern of discrete transmural fibrosis, which perfectly matches with the lesion revealed by the electroanatomical map (right). The intracardiac echo fan acquired during the mapping procedure is visualized in the map with the corresponding view in the small box. *Lower panels.* The planning target volume in the axial (**E**), coronal (**F**), and sagittal (**G**) view, as delineated by the red contour line, is depicted. The colored area represents the dose coverage, from the 95% to the maximum of the prescription dose. *Abbreviation:* CT, computed tomography

As previously described [16, 19], cardiac CT was performed using a whole-heart-coverage CT scan (Revolution CT, GE Healthcare): a first CT scan was obtained at the angiographic phase as routinely performed for coronary CT angiography, and a second delayed series of breath-hold and ECG-gated images was acquired for the detection of myocardial DE (Fig. 2, upper panel). Myocardial wall thinning (< 5 mm) and DE involving > 50% of myocardial thickness identified transmural DE. Dedicated post-processing reconstructions were applied to extrapolate single DICOM files for subsequent integration with EAM and with simulation-CT scans for SBRT target volume identification.

#### Electroanatomical mapping

High-density EAM was obtained with the CARTO system (Biosense Webster, Inc.); when possible, both an endo- and an epicardial LV map were used for an accurate 3D characterization of the scar and to facilitate CT image integration. Pre-acquired DICOM files were elaborated and merged by imaging fusion with EAM to characterize the VT substrate and validate the correspondence between diseased myocardium identified by EAM and fibrosis revealed by CT (Fig. 2, central panel and Movie 1 in the Supplementary data)*.* EAM was not included in patients with a contraindication to any interventional procedure in whom SBRT was guided by non-invasive imaging only.

#### Treatment plan preparation and radioablation session

The treatment plan was prepared by an experienced panel that involved two electrophysiologists, one clinical cardiologist, one cardio-radiologist, one biomedical engineer, two radiation oncologists, and two medical physicists, achieving a consensus on the target area, identified based on cardiac CT and EAM analysis, also considering all additional ECG and ECGI information.

A simulation free-breathing CT, a cardiac CT with contrast medium, and a “breathing-triggered” 4D-CT were performed and imported into the Eclipse Varian Treatment Planning System (Varian Medical System) to identify the following: (1) on the free-breathing CT, through a semi-automatic fibrosis localization (Fig. 1S), the target scar representing the clinical target volume (CTV), the surrounding organs at risk (OARs), and the ICD; (2) both on free-breathing CT and on 4D-CT, the heart in order to evaluate any displacement during the breathing cycle and to expand the CTV to create the internal target volume (ITV), taking into account heart respiratory motions. The planning target volume (PTV) was built further expanding the ITV in three dimensions considering residual uncertainties caused by patient positioning and movements, and by any displacement due to the heartbeat alone. On the free-breathing CT, a volumetric modulated arc therapy (VMAT) treatment plan was processed by Eclipse RapidArc Planning System (Varian Medical System), in order to deliver a total dose of 25 Gy in a single fraction (Fig. 2, lower panel) using the Varian Trilogy linear accelerator (Varian Medical Systems) with the patient in the immobilization cast. Patient set-up positioning was verified and corrected, if necessary, performing two cone-beam CT (image-guided radiotherapy, IGRT).

### Pre-discharge evaluation and management

After SBRT, all patients were continuously monitored for at least 5 days; routine laboratory tests and echocardiography examination were provided within 24 h after SBRT and before hospital discharge. All patients received dexamethasone plus additional gastro-protective agents for 20 days starting on the procedure day. ICD interrogation was performed immediately after SBRT and before patient discharge to verify device and lead parameters and exclude malfunctions. ICD VT detection and therapy parameters were maintained the same as before SBRT; sustained VT below the TDI were computed by the analysis of “VT monitored” and “ventricular sensed” events, after appropriate ICD reprogramming. ICD remote monitoring was set up for all patients.

### Follow-up

FU comprised weekly visits for the first month after SBRT and regular in-hospital controls at 3, 6, and 12 months after SBRT, including 12-lead ECG and ICD interrogation, echocardiography examination, and SF-36 Health Questionnaire. A radiotherapy evaluation and a thorax CT were planned at 3 and 12 months.

### Statistical analysis

Continuous variables were expressed as mean ± standard deviation and discrete variables as absolute numbers and percentages. The primary efficacy endpoint (reduction of ventricular arrhythmic episodes) and secondary endpoints (clinical, echocardiographic, and psychometric characteristics) were evaluated with the Student’s *T* test for paired data (continuous variables with normal distribution) or with the Wilcoxon sign rank test (variables with non-normal distribution). All tests were two-tailed and a *p* < 0.05 was considered significant.

## RESULTS

### Patients

Among 127 patients (pts.) with structural heart disease admitted for VT ablation at our Center between September 2019 and October 2020, 8 pts. (males, mean age 71 ± 8 years.; mean LVEF 27 ± 10%; 4 with ischemic cardiomyopathy (ICM), 4 with non‐ischemic cardiomyopathy (NICM)) were enrolled in the study, with alternative treatment options being excluded (Table 1). Seven pts. (mean age 70 ± 7 years) underwent SBRT: pt. #5, showing after enrollment a progressive hemodynamic decompensation due to intractable incessant VTs, was for this reason excluded from the study and eventually underwent an emergency endo-epicardial extracorporeal membrane oxygenation–supported percutaneous RFCA. Three of seven pts. had ICM, 4 pts. had NICM, and mean LVEF was 27 ± 11%. All patients presented with recurrent VT refractory to antiarrhythmic drugs (AADs) conditioning multiple ICD interventions; 4 pts. (#3, #4, #6, #8) presented with ES, 3 pts. (#1, #2, #7) had a coexistence of iterative VT below the TDI.

**Table 1:** Patients’ baseline characteristics

	Patient #1	Patient #2	Patient #3	Patient #4	Patient #5	Patient #6	Patient #7	Patient #8	
Age (years)	72	72	72	59	81	76	61	78	71 ± 8
Underlying cardiomyopathy	ICM	NICM	NICM	NICM	ICM	ICM	ICM	NICM	4 (50%) ICM 4 (50%) NICM
NYHA class	III	III	III	II	III	III	III	II	I - 2 (25%) II 6 (75%) III
LVEF (%)	23.5	21.1	20.3	44.4	20.4	20.7	20.8	41.3	27 ± 10
Device implanted	CRT-D	CRT-D	CRT-D	DDD ICD	CRT-D	CRT-D	CRT-D	CRT-D	
Stage of COPD (GOLD)	IV	III	II	II	II	IV	II	III	I - 4 (50%) II 2 (25%) III 2 (25%) IV
CKD stage	Severe	Severe	Severe	Mild	Mild	Severe	Mild-moderate	Mild-moderate	
BMI (kg/m^2^)	30.46	24.72	30.06	25.47	20.76	23.88	33.75	25.35	26.81 ± 4.23
Disthyroidism	Hyper-	Hypo-	Hypo-	No	No	No	No	No	
Atrial fibrillation	Paroxysmal	Permanent	Permanent	No	No	No	No	Persistent	
Arrhythmia presentation	VT, NIVT	VT, NIVT	VT (ES)	VT (ES)	VT (ES)	VT (ES)	VT, NIVT	VT (ES)	
Prior cardiac surgery	Yes	Yes	Yes	No	No	Yes	No	Yes	
Clinical peculiarities	Mitral-clip	Mitro-aortic mechanical prosthesis, cardiac-support device	Surgical CRT-D, LA thrombus, scar in proximity to LAD	Severe anemia, scar in proximity to LAD	–	LV mobile thrombus, ACBPG, pericardiectomy	Severe systemic arteriopathy	Mitral annuloplasty with severe residual insufficiency, aortic bioprostheses, bladder neoplasm	
Ongoing AAD (N)	2	3	3	3	3	3	3	2	
Previous VT catheter ablations (N) endo/epi	3/2	0	1/0	2/1	1/0	0	3/0	2/0	

*AAD* antiarrhythmic drugs, *ACBPG* aortocoronary bypass graft, *BMI* body mass index, *CKD* chronic kidney disease, *COPD* chronic obstructive pulmonary disease, *CRT-D* cardiac resynchronization therapy defibrillator, *DDD ICD* dual-chamber implantable cardioverter defibrillator, *ES* electrical storm, *ICD* implantable cardioverter defibrillator, *ICM* ischemic cardiomyopathy, *LA* left atrium, *LAD* left anterior descending artery, *LVEF* left ventricle ejection fraction, *NICM* non-ischemic cardiomyopathy, *NIVT* near-incessant ventricular tachycardia, *NYHA* New York Heart Association, *VT* ventricular tachycardia

Five pts. had received a previous EAM LV mapping (endo- and epicardial in two pts.); 2 pts. were unsuitable for any electrophysiological mapping (pt. #2 had mitral-aortic mechanical prosthesis and a cardiac-support device; pt. #6 presented with a mobile LV thrombus detected by CT and had undergone aortocoronary bypass graft plus pericardiectomy). In pt. #2, VT ECGI during non-invasive electrophysiological study was obtained.

### Procedural outcome

Treatment characteristics for each patient are depicted in Table 2. No procedural adverse effect was observed.

**Table 2:** Treatment characteristics

	Patient #1	Patient #2	Patient #3	Patient #4	Patient #6	Patient #7	Patient #8	
Tools used for target scar definition	Endo-epi EAM, CT, ECG	CT, ECGI, ECG	Endo EAM, CT, ECG	Endo-epi EAM, CT, ECG	CT, ECG	Endo EAM, CT, ECG	Endo EAM, CT, ECG	
Target scar location	Infero-postero-lateral	Basal perivalvular	Subepi basal anteroseptal (close to LAD)	Subepi mid-basal anteroseptal (close to LAD)	Anteroseptal, apex (LV aneurysm)	Anteroseptal, apex (LV aneurysm)	Basal infero-postero-lateral	
Clinical target volume (cm^3^)	43.7	16.04	55	14.1	41.1	53.35	51.4	39 ± 17
Internal target volume (cm^3^)	115.9	54.4	120.9	93.9	131.8	145.5	116.4	111 ± 30
Planning target volume (cm^3^)	198.3	88.1	187.6	138.7	225.3	239	203.7	183 ± 53
*D*_95%_ (%)	94.9	96.2	94.6	97	94.9	95	96.1	96 ± 1
*V*_95%_ (%)	94.8	97	94.1	98.3	94.5	95	97.2	96 ± 2
Treatment time (min)	25	22	28	24	21	27	37	31 ± 6

*CT* computed tomography, *EAM* electroanatomical mapping, *ECG* electrocardiography, *ECGI* non-invasive electrocardiographic imaging, *LAD* left anterior descending artery, *LV* left ventricle

**Fig. 3:**
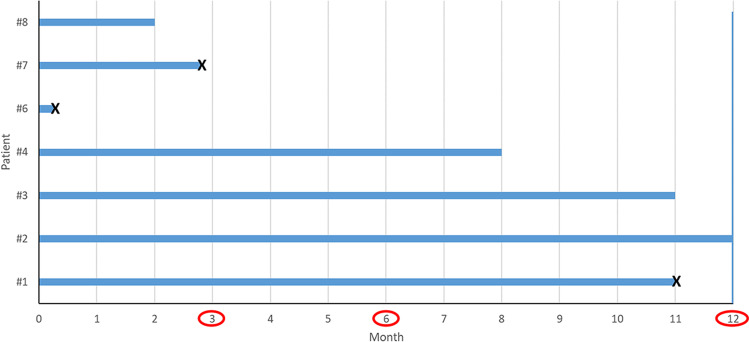
Follow-up summary. Months after SBRT for each patient are depicted, no patient was lost on follow-up and 4 patients completed the 6-month follow-up period. Red circles highlight the time for in-hospital check-ups; cross indicates patient’s death. *Abbreviation:* SBRT, stereotactic body radiotherapy

At a median FU of 8 months (Fig. 3), 3 pts. died: pt. #1 died 11 months after SBRT because of worsening heart failure; pt. #6 in the post-SBRT hospitalization phase because of multiorgan failure following bacterial pneumonia and sepsis, in the absence of arrhythmia recurrence. Patient #7 was found dead at his home during the third month after SBRT, and no reason for death was found. The patient died few days before the 3-month FU visit, but he was strictly followed up because of symptomatic VT episodes below the TDI recurring in the early post-ablation phase, episodes that completely resolved 2 months after SBRT. Even if an arrhythmic event was not the cause of death, a cardiac *non-arrhythmic* death in this patient cannot be excluded, as no autopsy was performed. Four patients fulfilled the 6-month FU program. All pts. maintained continuous ICD remote monitoring. In pt. #2 and pt. #4, amiodarone was down-titrated to 800 mg/week, starting 4 months after SBRT; in pt. #3, mexiletine was permanently withdrawn less than 1 month after SBRT.

#### Safety

No acute toxicity and no adverse effect with ICD were observed during SBRT and during early post-SBRT phase. During the entire FU period, no treatment-related SAE occurred. Pt. #1, in whom the CTV was very close to the stomach, complained of nausea and vomiting for few days after SBRT and was effectively treated with additional antiemetic drugs. In patient #4, the 6-month CT scan showed a small area of paramediastinic pulmonary fibrosis in the absence of symptoms or hypoxia. Table 3 shows the complete list of adverse events.

**Table 3:** Adverse events

Event	Patient	Grade (1–5)	Correlation with SBRT	Time of apperance (month)
Nausea	1 patient (P01)	2	Definite	1
Gastrointestinal disorders	1 patient (P01)	2	Definite	1
SARS-CoV-2	1 patient (P03)	3	Unrelated	1
Vertebral fracture	1 patient (P03)	3	Unrelated	5
Pulmonary fibrosis	1 patient (P04)	1	Definite	4
Cardiac death (heart failure)	1 patient (P01)	5	Unrelated	11
Non-cardiac death (sepsis)	1 patient (P06)	5	Unrelated	1
Unexplained death	1 patient (P07)	5	Not evaluable	3

#### Efficacy

Four patients (pt. #1, pt. #2, pt. #3, pt. #4) have completed the 6-month post-procedural FU; pt. #8 has just entered the third follow-up month, had no VT recurrence, but is not included in the analysis.

**Fig. 4:**
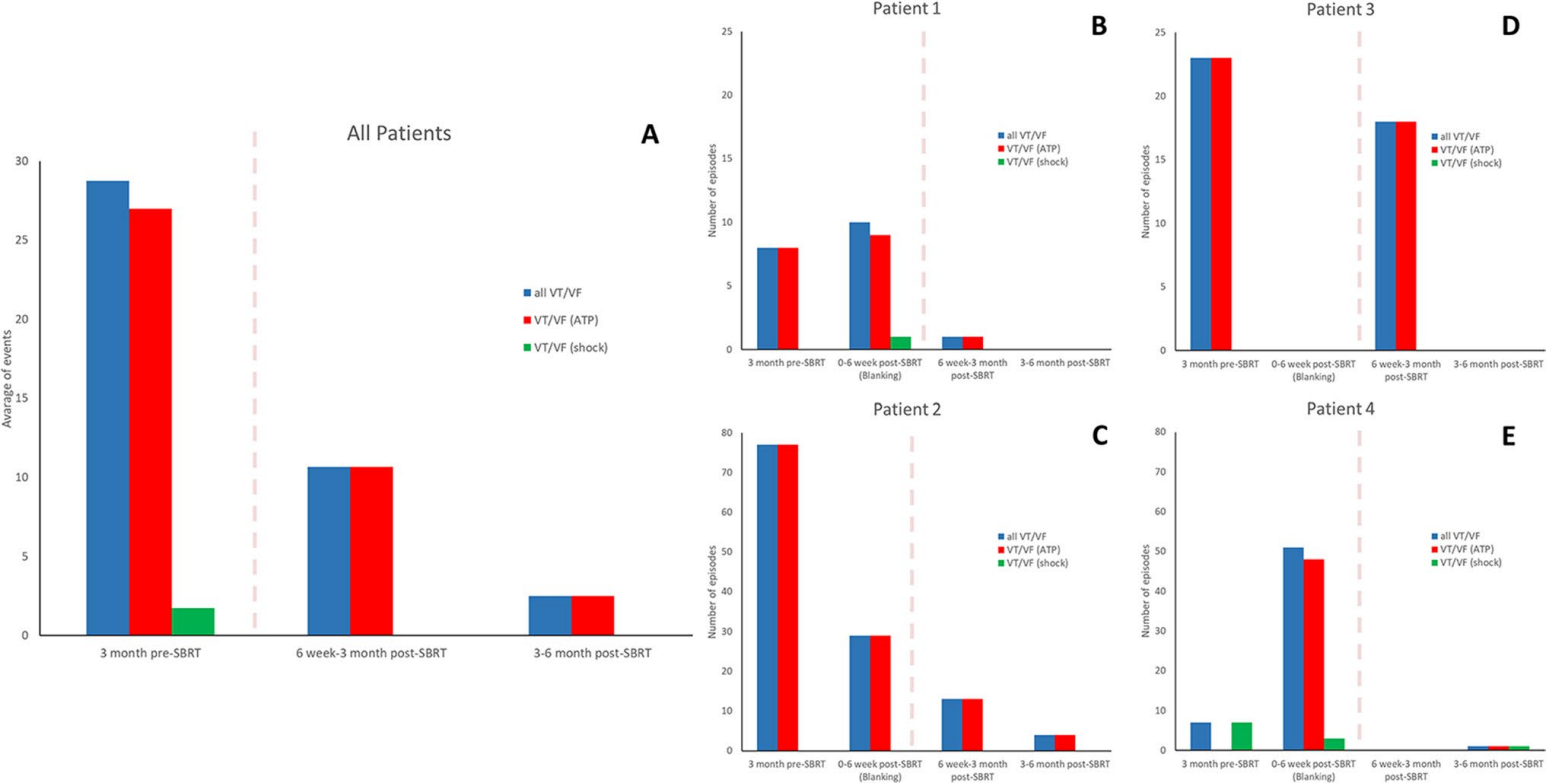
Outcome of SBRT for the patients having completed the 6-month follow-up. From left to right, the number of VT episodes occurring during the 3 months preceding SBRT and during follow-up (up to 3 months from SBRT and in the following 3-month period) is depicted by bars. Blue bars refer to all VT episodes, red bars to VT causing ATP, green bars to VT causing shock. **A** Cumulative number of VT episodes, as average of events. **B**, **C**, **D**, and **E** Absolute number of VT episodes for each patient. *Abbreviations:* ATP, antitachycardia pacing; SBRT, stereotactic body radiotherapy; VT, ventricular tachycardia

**Fig. 5:**
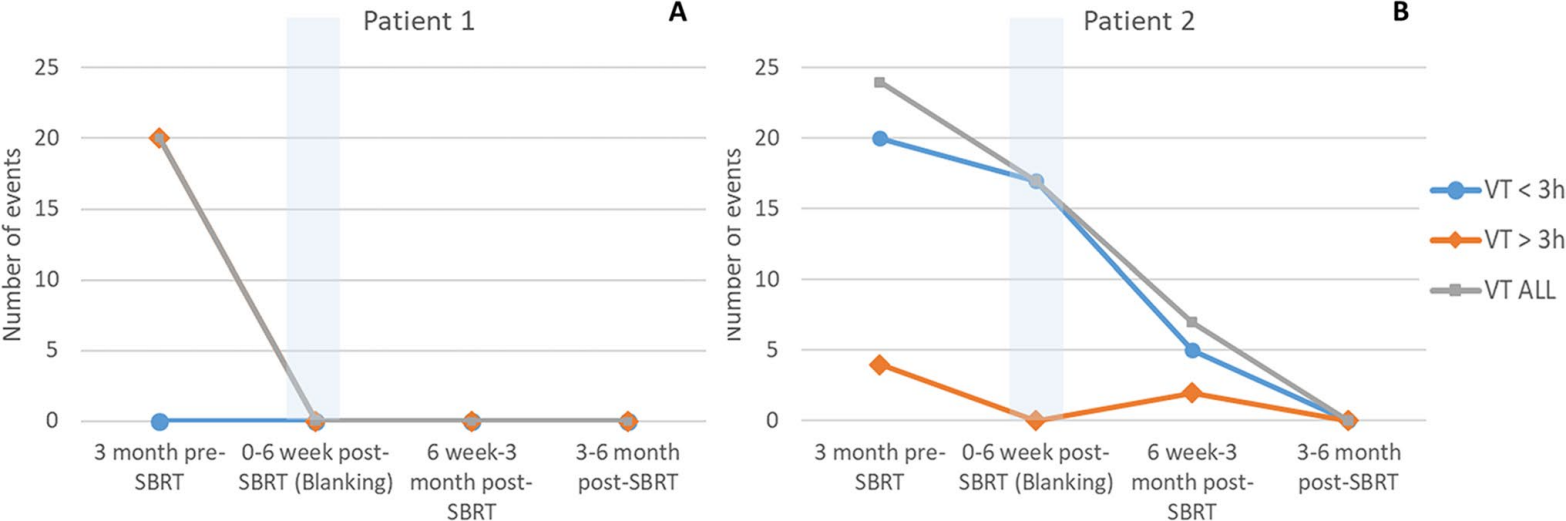
Outcome in patients with iterative slow VTs, not accounting for ICD interventions. The numbers of VT episodes during the 3 months preceding SBRT and during follow-up (up to 3 months from SBRT and in the following 3-month period) for pt. #1 (**A**) and for pt. #2 (**B**) are represented. Gray dots and lines refer to the overall number of VTs, orange to VT episodes lasting > 3 h, light blue to VT episodes lasting < 3 h. An early favorable trend is observed in these patients starting from the «blanking period». *Abbreviations:* SBRT, stereotactic body radiotherapy; VT, ventricular tachycardia

In comparison with the 3-month period preceding SBRT, the total mean number of VT episodes decreased from 29 ± 33 to 11 ± 9 at 3 months (*p* = 0.05), and to 2 ± 2 at 6 months (*p* = 0.08) (Fig. 4A). The number of VT episodes with ATP was reduced from 27 ± 35 to 11 ± 9 (*p* = 0.05) and to 2 ± 2 (*p* = 0.2), at 3 and 6 months, respectively; the number of VT episodes with shock was reduced from 2 ± 3 to 0 (*p* = n.s.) and 0 (*p* = 0.13), at 3 and 6 months, respectively. The number of appropriate shocks decreased from 11 to 0 and to 2, at 3 and at 6 months, respectively. Three out of four patients showed a substantial reduction of VT episodes and ICD interventions at 3 and 6 months (Fig. 4B, C, D, E). Pt. #3 contracted shortly after SBRT a serious COVID-19-related pneumonia necessitating prolonged respiratory assistance and full steroid therapy; as already mentioned, mexiletine was stopped at this time. The patient partially recovered approximately 4 months after SBRT; an impressive clearing from arrhythmias was achieved contextually in this patient in the second 3-month FU period (Fig. 2S). In pt. #1 and pt. #2 also the complete remission of the VTs below TDI was observed (Fig. 5), with restoration of stable cardiac resynchronization. No patient presented with ES or underwent subsequent RFCA; all 4 pts. are in good condition at the time of paper submission.

Given the few patients enrolled so far, analysis of mortality was not performed. At the 3- and 6-month FU, LVEF changed from 29 ± 10% to 28 ± 5% (n.s.) and 32 ± 5%, (n.s.), respectively. The SF-36 QoL questionnaire showed at 6 months a global slight improvement in the items concerning physical functioning (16 to 35), role physical (25 to 38), health perception (37 to 52), and vitality (34 to 53), without univocal changes in the mental component summary (Fig. 3S).

## Discussion

Preliminary results of the phase Ib/II STRA-MI-VT study show that SBRT, applied as a bailout for VT in patients with severe cardiomyopathy, is feasible and presents a high safety profile. Efficacy in VT prevention has been demonstrated in the whole cohort over a middle-term FU.

To the best of our knowledge, STRA-MI-VT is the first European prospective study investigating the role of SBRT for the treatment of malignant arrhythmias. Due to the small number of patients enrolled, our current findings have a limited statistical power but convincingly promote the curative role of this novel technique, which has the prerequisites to be further investigated in selected groups of patients.

### Main findings

In our study, SBRT was addressed to patients with severe cardiac disease and an unconditioned indication for the treatment of recurrent VTs, not eligible to any other therapy option. Results on safety have been acquired over the mid- to long term in 4 patients (#1, #2, #3, #4) with reassuring evidences, in the absence of adverse effects. Among the 3 additional patients (#6, #7, #8) in whom data is available from the short-term period only, two deaths occurred: one (pt. #6) just after SBRT and clearly unrelated to the treatment and the other (pt. #7) completely unexplained but not related to arrhythmic causes. No SBRT-related relevant complication was observed.

On the other hand, favorable effects of SBRT on ventricular arrhythmias have been observed in all patients on follow-up, while maintaining AADs unchanged or slightly reduced in selected cases. In fact, pts. #1, #2, and #4 showed the substantial regression of the arrhythmia burden after completion of the blanking period, with a clear reduction in VT episodes and the absence of ES, at 3-month and 6-month follow-up. In pt. #3 the regression of the VT burden was postponed to the second 3-month FU period: one may speculate that severe acute comorbidities may have impacted on the early outcome of this patient. Moreover, in pts. #1 and #2, the clear-cut evidence of an early remission of iterative VTs confirms the particularly favorable effect of SBRT on slow VT circuits [15]. As a consequence, overall QoL showed a perceivable improvement, impacting as reasonable mainly on physical well-being: given both the very small sample size and the observed high intrasubject variability, a larger sample is obviously required to predict the effects of SBRT on the patients’ short- and long-term quality of life.

### Analysis of previous experiences

In the clinical setting, starting from the leading experience of Cuculich and Coll. [11], data on SBRT referred to single case reports or small patient series [14], while one retrospective observational study comprising 10 pts. showed a favorable outcome over the long term in patients treated with CyberKnife [13]. Currently, only two prospective US studies [14, 15] have investigated the potential role of SBRT in patients with ventricular arrhythmias. From the ENCORE-VT Study, Robinson and Coll. [15] reported the results of a series of 19 pts. with ventricular arrhythmias and either structural heart disease or “premature ventricular complex”-induced tachycardiomyopathy, in whom SBRT determined the significant reduction of the arrhythmia burden in 94% of patients with a discrete incidence of serious adverse events (pericarditis, 28%; pneumonitis, 11%). At a 12-month follow-up, 6 patients died, 3 for cardiac reasons. Gianni and Coll. [14] reported the results over the long-term follow-up in 5 patients undergoing SBRT using the CyberKnife: in the face of good outcomes in the early phase, a high probability of recurrences over the mid- to long term was noted, and, at a mean FU of 12 months, 2 patients died of advanced heart failure. Interestingly, smaller PTV, but also a more invasive approach and longer procedural times were noted with the use of CyberKnife. In both studies, no SBRT-related death was identified, but the overall survival at 1 year ranged from 60 to 72% with a prevalence of cardiac death. In our population of patients with advanced cardiac disease and a bailout indication for SBRT, 2 patients died (intractable heart failure after 1 year and unexplained death at 3 months), showing a comparable outcome in terms of cardiac mortality.

### Technical issues

Delayed enhancement cardiac CT was the imaging of choice for LV scar characterization in the present study. Extra-coronary application of cardiac CT is gaining a pivotal role in cardiac imaging, and previous data [19, 20] supported the use of cardiac CT for myocardial fibrosis identification. Of most importance, a last-generation CT scan combined with a dedicated algorithm in the post-processing phase was employed to enhance accuracy in scar detection [19]. Cardiac CT imaging was imported directly on the simulation CT and eventually merged with 4D CT, thus improving the accuracy of target area identification and translation into the treatment plan. This process may have contributed to optimize the effectiveness of SBRT and to limit radiation toxicity; more specifically, we disregarded the use of cardiac magnetic resonance imaging, which is widely taken as the gold standard for myocardial fibrosis evaluation [19], considering the characteristics of this very selected group of patients, in whom the presence of an ICD and the arrhythmic presentation would significantly impact accuracy and safety of this technique.

### Expectations and concerns

SBRT was applied to a selected cohort of patients in whom SBRT was the only feasible option, as an extreme bailout, based on clinical characteristics and severity of the underlying cardiomyopathy.

Theoretical disadvantages refer, however, to the fact that this novel therapeutic approach has been tested only in selected groups of patients and there is no current evidence of its efficacy as a standard treatment. Moreover, differently from conventional RFCA procedures, given the complex lesion maturation process caused by SBRT, therapeutic effects develop within a few months [12, 15]. This evidence was confirmed in our experience and may restrict the indications for its use in unstable patients.

At this time, our study reasonably confirms the absence of SBRT-related SAEs, over a median 8-month FU. Even if preclinical studies support the hypothesis that lesion formation is achieved within a maximum of 6 months, SAEs over a longer time frame cannot be completely excluded, underlining the need for longer surveillance. Equally, the potential risk for late radiation–induced lesions deserves great caution and may limit the use of SBRT in young patients.

Thus, we stand in favor of larger controlled studies where strict enrollment criteria and a rigorous methodology must be adopted in order to guarantee a favorable risk–benefit ratio for this still investigational form of treatment.

Further investigation may contribute to optimize cost-effectiveness, with particular respect to imaging and treatment modalities, including doses, delivery, and regimens in the radioablation setting. Currently, the diagnostic and therapeutic workflow required for SBRT is complex and requires a thorough cooperation among different medical and technical figures with specific competences in the field of electrophysiology, cardiac imaging, and radiation therapy.

### Limitations

This is a non-randomized study conducted at a single center; moreover, results are preliminary and refer to a small number of patients and a relatively short FU. For these reasons, further evaluation is required, with respect to effectiveness but also to safety, particularly over the long term.

## Conclusions

Preliminary results of the STRA-MI-VT Study demonstrate a favorable cost-effectiveness ratio of SBRT, in the absence of major adverse events over the mid- to long term. Further investigation in patients with structural heart disease not suitable for conventional options of treatment will help in clarifying the effective role of SBRT in light of the attractive possibility to treat arrhythmia substrates that are inaccessible to conventional approaches in a safer and less-invasive manner.

## Data and material availability

Data were deposited in the ZENODO repository (doi: 10.5281/zenodo.5547476).

## Supplementary Information

Below is the link to the electronic supplementary material.

Supplementary file2 (DOCX 636 kb)

Supplementary file1 (MP4 27336 kb)

## Data Availability

Not applicable.

## Abbreviations

AAD: Antiarrhythmic drugs
ACBPG: Aortocoronary bypass graft
ATP: Antitachycardia pacing
BMI: Body mass index
CKD: Chronic kidney disease
COPD: Chronic obstructive pulmonary disease
CRT-D: Cardiac resynchronization therapy defibrillator
CT: Computed tomography
CTCAE: Common Terminology Criteria for Adverse Events
CTV: Clinical target volume
DDD ICD: Dual-chamber implantable cardioverter defibrillator
DE: Delayed enhancement
EAM: Electroanatomical mapping
ECG: Electrocardiography
ECGI: Non-invasive electrocardiographic imaging
EF: Ejection fraction
ES: Electrical storm
FU: Follow-up
ICD: Implantable cardioverter defibrillator
ICM: Ischemic cardiomyopathy
IGRT: Image-guided radiotherapy
ITV: Internal target volume
LA: Left atrium
LAD: Left anterior descending artery
LV: Left ventricle
NICM: Non-ischemic cardiomyopathy
NIVT: Near-incessant ventricular tachycardia
NYHA: New York Heart Association
OAR: Organ at risk
PTV: Planning target volume
QoL: Quality of life
RFCA: Radiofrequency catheter ablation
SAE: Serious adverse event
SBRT: Stereotactic body radiotherapy
TDI: Tachycardia detection interval
VA: Ventricular arrhythmia
VF: Ventricular fibrillation
VMAT: Volumetric modulated arc therapy
VT: Ventricular tachycardia
