# Supplementary material for: Stereotactic radioablation for the treatment of ventricular tachycardia: preliminary data and insights from the STRA-MI-VT phase Ib/II study
Source: J Interv Card Electrophysiol. 2021 Oct 5;62(2):427–39. doi: 10.1007/s10840-021-01060-5 (PMC8490832; doi:10.1007/s10840-021-01060-5)
Supplement: Supplementary file 1 — Supplementary file2 (DOCX 636 kb) [file 10840_2021_1060_MOESM1_ESM.docx]

**SUPPLEMENTARY DATA**

**METHODS**

***Cardiac computed tomography.***

Cardiac CT was performed using a whole heart coverage CT scan (Revolution CT, GE Healthcare, Milwaukee, WI, USA) with the following parameters: slice configuration 256x0.625mm, gantry rotation time 280ms and prospective electrocardiography (ECG) triggering. Patients received a 1.5ml/kg bolus of contrast medium (Iomeron 400mg/mL, Bracco), followed by saline infusion. A first CT scan was obtained at the angiographic phase to have adequate coronary artery contrast opacification as routinely performed for coronary CT angiography. A second series of breath-hold and ECG-gated images was acquired after 8 minutes from contrast agent injection for the detection of myocardial DE (**Fig 2**, upper panel). Visual evaluation of DE was performed and a narrow window width and level (350W and 150L) was used for late scan evaluation that is best viewed as thick average intensity projections (5-8mm). Myocardial wall thinning (wall thickness <5mm) and DE involving >50% of myocardial thickness was considered transmural DE. A dedicated post-processing reconstruction was applied to extrapolate single DICOM files including only myocardial fibrosis volume, coronary and ascending aorta anatomy, pericardial fat volume to be merged with EAM. Eventually, a second dedicated DICOM series including myocardial fibrosis was prepared for subsequent integration in the simulation CT for SBRT target volume identification.

***Electroanatomical mapping.***

High-density EAM was obtained with the 3D-CARTO system (Biosense Webster, Inc., Diamond Bar, CA, USA), using the Pentaray or Decanav catheter (Biosense Webster, Inc.); when possible, both an endo- and an epicardial LV map were used for an accurate 3D-characterization of the scar and to facilitate CT image integration of all anatomical structures. Basically, cut-off values of bipolar endocardial voltage were 1.5mV for diseased myocardium and .05mV for “dense scar”, respectively. Unipolar endocardial and bipolar epicardial electrograms (EGMs) were considered pathological if amplitude was <8.0mV and/or <1.0mV, respectively. Pre-acquired DICOM files were elaborated and merged by imaging fusion with EAM to characterize the VT substrate and validate the correlation between diseased myocardium identified by EAM and fibrosis revealed by CT (**Fig 2**, central panel and **Movie 1**)*.* It was accepted not including EAM in selected patients with a contraindication to any interventional procedure in whom SBRT was guided by non-invasive imaging only.

***Treatment plan preparation and radioablation session.***

The treatment plan was prepared by an experienced panel that involved two electrophysiologists, one clinical cardiologist, one cardio-radiologist, one biomedical engineer, two radiation oncologists and two medical physicists, achieving a consensus on the target area, identified based on cardiac CT and EAM analysis, also considering all additional ECG and ECGI information.

The patient was first positioned in supine orientation into an immobilization cast and underwent a simulation free breathing CT (2.5mm slices). A cardiac CT with contrast medium and a “breathing-triggered” 4D-CT were then acquired and imported together with the simulation CT into the Eclipse Varian Treatment Planning System (Varian Medical System, Palo Alto, CA, USA) with the aim to identify:

*1)* the target scar representing the clinical target volume (CTV) and all surrounding anatomical structures, also known as organs at risk (OARs), including the ICD. In particular, to identify the CTV, the simulation CT was merged with the CT DICOM series using main pulmonary artery and its primary branches as 3D references in order to obtain a semi-automatic fibrosis location (**Fig 1S**). Through a rigid fusion between the cardiac CT and the free-breathing CT, all the OARs were finally reported on the free-breathing CT.

*2*) the heart, both on free breathing CT and on 4D-CT, in order to evaluate its displacement during the breathing cycle and to quantify the margins to apply to the CTV so to create the internal target volume (ITV), taking into account heart respiratory motion.

Starting from ITV, a planning target volume (PTV) was built, expanding the ITV in three dimensions by a margin of 3 mm, related to any residual uncertainty due to patient positioning and movements and any other displacement due to the heartbeat alone**.** On the free breathing CT, a volumetric modulated arc therapy (VMAT) treatment plan was processed by Eclipse RapidArc Planning System (Varian Medical System), in order to deliver a total dose of 25Gy in a single fraction (**Fig 2**, lower panel). The VMAT treatment was performed by using two to four arc beams and SBRT was delivered using the Varian Trilogy linear accelerator (Varian Medical System) on the in-patient basis. The procedure was carried out with the patient in a comfortable position in his/her immobilization cast. Before and during the treatment, the patient set-up positioning was verified and corrected, if necessary, performing two cone-beam CT (image-guided radiotherapy, IGRT).

**Fig 1S Merging process between the free-breathing simulation CT and the CT for myocardial fibrosis detection.**

The main pulmonary artery (green contour) and its primary branches represent the tridimensional landmark (derived from CT for myocardial fibrosis) to achieve accurate matching between the two acquisition modes; myocardial fibrosis (green contour) is matched on the simulation CT in a semi-automatic way. Three panels represent axial, coronal and sagittal view of merged CT data.

**
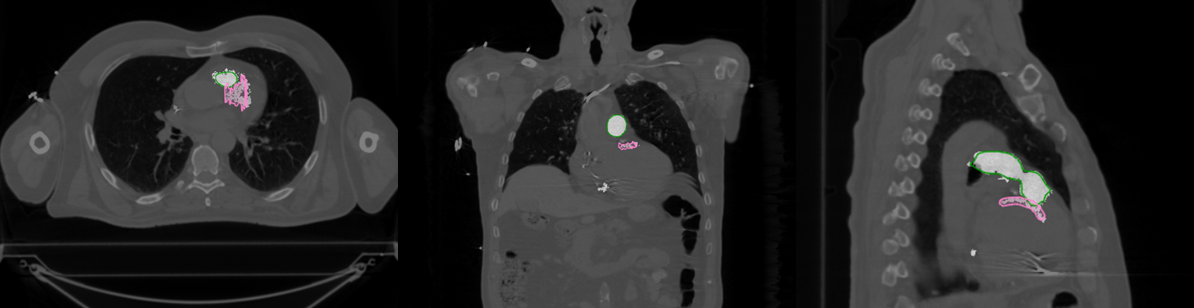
**

*Abbreviation:* CT: Computed Tomography

**Fig 2S Pt. #3 procedural outcome.**

Number of VT episodes during the entire follow-up period is depicted. Light blue points identify the cumulative number of VT for each month, from the 3 month period preceding SBRT to the most recent follow-up. Despite an immediate favorable response, frequent recurrences occurred in this patient during the 2^nd^ and 3^rd^ month after SBRT, in concomitance with a severe COVID19 disease. A dramatic reduction of patient’s VT burden (evidenced by the light blue line) was observed starting from the 4^th^ follow-up month, as **the patient progressively recovered from his disease. A permanent control of recurrences was obtained during the subsequent follow-up, despite withdrawal of mexiletine.**

*
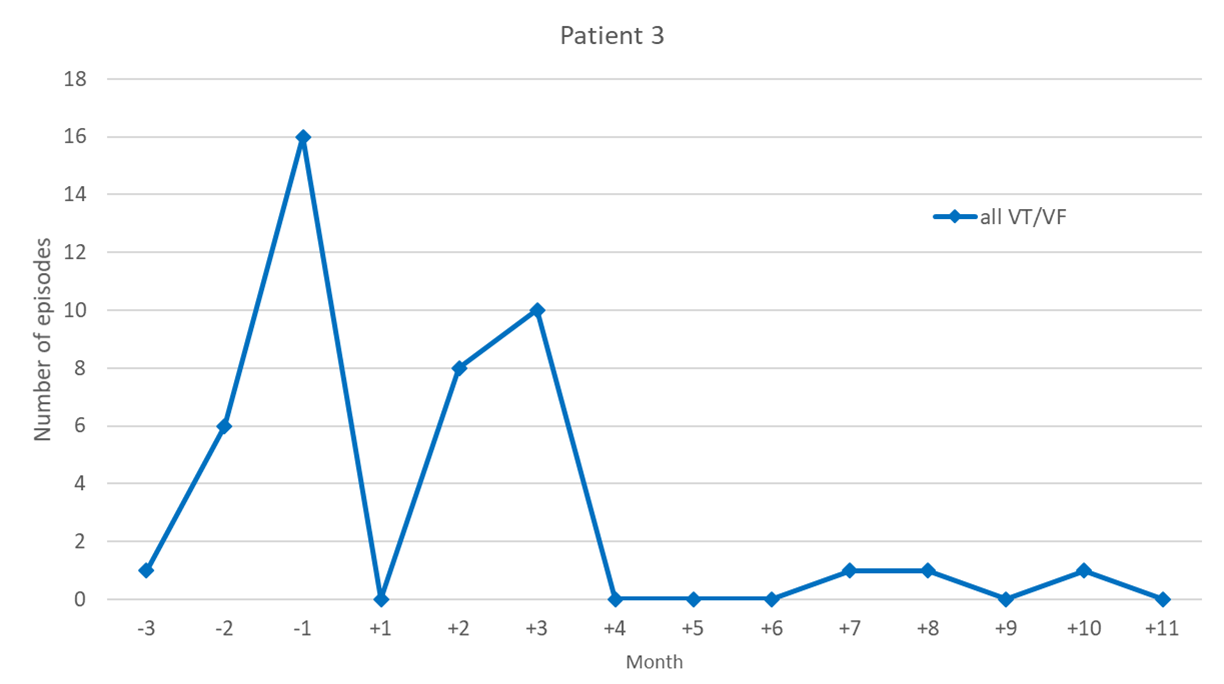
 Abbreviation:* SBRT: Stereotactic Body Radiotherapy, VT: Ventricular Tachycardia

**Fig 3S The 8 bar graphs represent the mean scores for each SF-36 domain.**

Blue bars are related to physical well-being, light blue bars to psychological well-being. SF-36 was administered at baseline (pre-SBRT), at 3 and at 6mth follow-up. Mean scores of the SF-36 are represented on the Y axis, ranging from 0 to 100, respectively the worst and the best score for each domain.

QoL showed an improvement from baseline at 3 and 6mth in **physical functioning** (baseline: **16**; 3mth: **29**; 6mth: **35**), **health perception** (baseline: **37**; 3mth: **42**; 6mth: **52**) and **vitality** (baseline: **34**; 3mth: **52**; 6mth: **53**). Conversely, the **emotional role functioning** showed a constant aggravation from the baseline to the 6mth follow up (baseline: **58**; 3mth: **44**; 6mth: **42**); with respect to the remaining domains we observed a decrease followed by an increase in **physical role functioning** (baseline: **25**; 3 mo.: **8**; 6 mo.: **38**), and minor changes in **bodily pain** values, **social role functioning,** and **mental health,** as depicted.


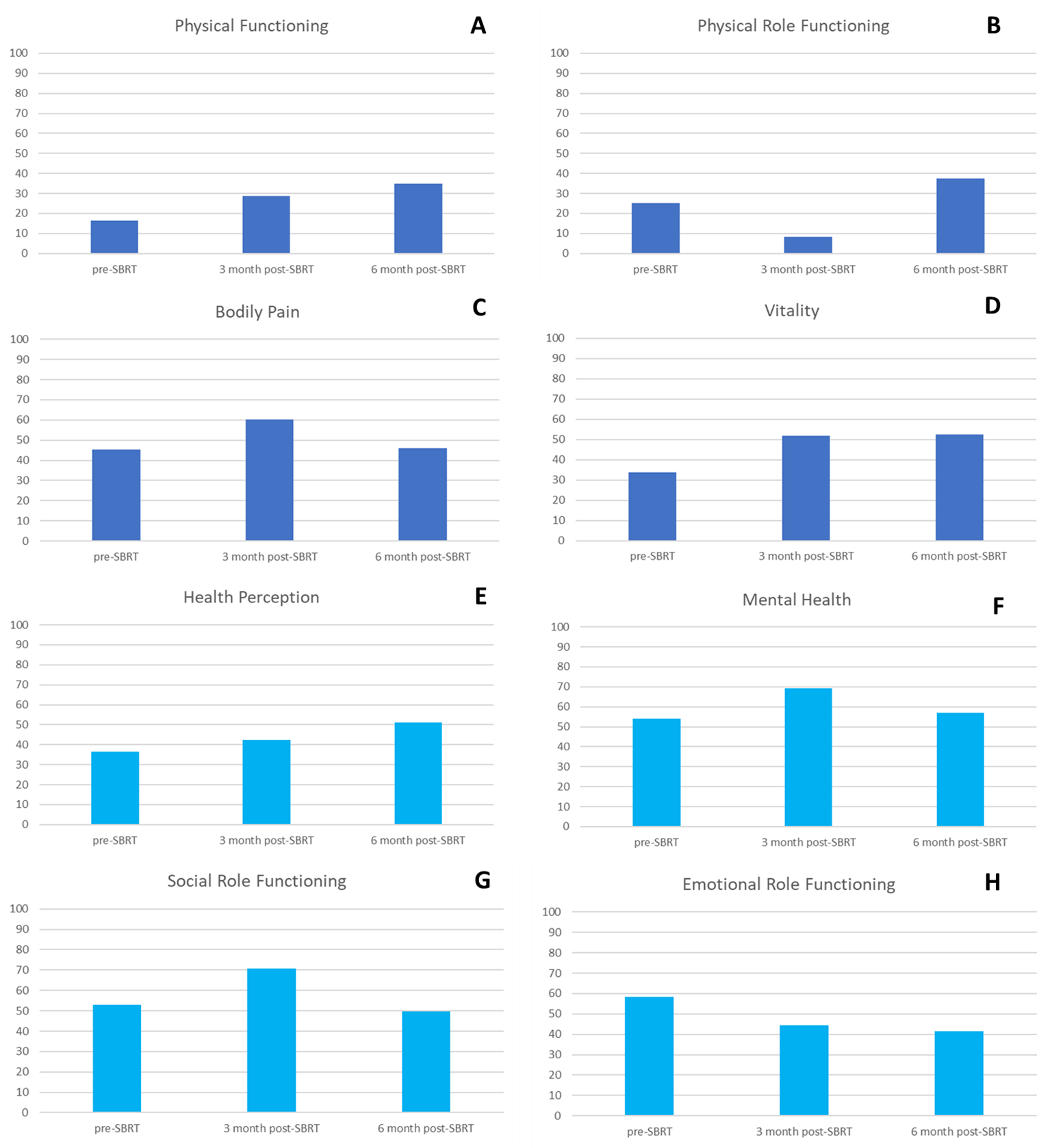


*Abbreviation:* QoL: Quality of Life, SBRT: Stereotactic Body Radiotherapy.

**Movie 1. Real-time integration of a CARTO 3D-electroanatomical map with cardiac CT, obtained by endo-and epicardial fusion.**

The anatomical match between the cardiac shell, elaborated by CT, and the epicardial contours, defined by high-density electro-anatomical mapping, is obtained by a customized semi-automatic algorithm and verified by the operator considering multiple anatomical markers, to reproduce with highest accuracy patient’s anatomy.

Once the images of the cardiac fibrosis have been imported in the electro-anatomical map, multiple views of the integrated map are used to visualize the extent and location of the myocardial fibrosis with regard to the different myocardial segments involved. Fibrosis detected by CT is compared with the diseased area of myocardium represented on the epicardial and on the endocardial map for an appropriate combined definition of the target area in a 3D model, as highlighted by the transparency. The area of diseased myocardium recognized by electro-anatomical mapping is represented, as usual, by a color-coded map.

The evidence, provided by CT, of a scar with transmural involvement in the infero-posterior wall of the left ventricle is confirmed by the analysis of the EGMs both on the epicardial as on the endocardial side (dense scar by red). Of note, EGMs characterized by reduced voltage amplitude are revealed also in correspondence of the major coronary vessels. The combined use of CT-imaging with electroanatomical mapping is considered of additional value to enhance global accuracy in the identification of the functional and structural diseased substrate to be considered the target for SBRT.

*Abbreviation:* CT: Computed Tomography, EGM: Electrograms, SBRT: Stereotactic Body Radiotherapy.
